# The role of patient reported symptom data in co‐producing meaning in rheumatoid arthritis

**DOI:** 10.1111/jep.14182

**Published:** 2024-10-13

**Authors:** Sarah Skyrme, William G. Dixon, Sabine N. van der Veer, Caroline Sanders, Charlotte A. Sharp, Dawn Dowding

**Affiliations:** ^1^ School of Health Sciences University of Manchester Oxford Road Manchester M13 9PL UK; ^2^ Division of Health Sciences Informatics, Imaging and Data Science University of Manchester Oxford Road Manchester M13 9PL UK; ^3^ Division of Musculoskeletal and Dermatological Sciences, School of Biological Sciences, Faculty of Biology, Medicine and Health The University of Manchester Manchester UK; ^4^ The Kellgren Centre for Rheumatology, Manchester Royal Infirmary Manchester University NHS Foundation Trust Manchester M13 9WL UK

**Keywords:** arthritis, rheumatoid, decision making, shared, mobile applications, patient generated health data, qualitative research, symptom assessment

## Abstract

**Rationale:**

Patients with rheumatoid arthritis (RA) experience a range of symptoms including joint pain and inflammation, stiffness, fatigue, anxiety, and low mood. Similar to patients with other long‐term conditions, they may have periods of time when their disease is under control, and times when their condition is less stable, requiring treatment adjustments. The REMORA2 feasibility study explored the implementation of an integrated symptom‐tracking system using a smartphone application (app), enabling patients to track day‐to‐day symptoms. The data was available in the electronic health record to be viewed at subsequent consultations.

**Aims and Objectives:**

This paper explores patients' comments on living with RA, and how patient‐reported symptom data supports informed interactions as patients and clinicians work together to coproduce meaning from the data.

**Method:**

Individual semi‐structured interviews were conducted with 21 patients and 7 clinicians, supplemented by nonparticipant observations of 5 clinical appointments. Thematic analysis was used to analyse data from the interviews, with an ethnographic approach used to analyse the observational data.

**Results:**

Both clinicians and patients reported the benefits of reviewing the data in the clinic together. This helped inform decisions about pain management and identified patients who might otherwise have dismissed symptoms such as pain, because of their natural inclination to be stoical.

**Conclusion:**

Improved insights on the care of RA were generated as patients and clinicians discuss symptom tracking data. This can assist the patient‐clinician dyad in the process of two‐way learning and shared decision‐making on the management of a long‐term condition.

## 1 INTRODUCTION

Patients with rheumatoid arthritis (RA) experience a range of symptoms including joint pain, stiffness, fatigue, anxiety, and the need for adaptations to their lifestyle, which can be challenging to adjust to.^1^, ^2^, ^3^ Life with RA can entail times when symptoms are under control, interspersed with periods of increased disease activity accompanied by joint inflammation, pain, worsened fatigue, poor sleep, and limited activity.^1^ These factors shape and impact wellbeing and life choices, such as employment, and are likely to influence dimensions of peoples' relationships with families, friends, and colleagues.^4^

Living with RA encompasses the individualised task of adapting to the presence of the condition and balancing medication regimes with the demands of life.^4^ Effective two‐way communication between the patient‐clinician dyad can support patients in making these adaptations and managing their condition.^1^ However, during consultations, patients may struggle to recall the details of their RA since their last appointment due, in part, to the fluctuating nature of symptoms and the challenge of summarising them during infrequent consultations.^5^, ^6^ This can be compounded if they feel they should be a ‘good’ patient who does not complain.^7^, ^8^

The Remote Monitoring of RA (REMORA) programme entails the design, evaluation, and implementation of an integrated symptom‐tracking system for people with RA.^9^ It consists of a smartphone app that gathers data on patients' disease activity, assisting recall of symptoms when they attend consultations. Patients input daily, weekly, and monthly data into the app, scoring pain, stiffness, fatigue and other symptoms. This data is integrated into the electronic health record and can be viewed by their clinician during consultations, providing a graphical overview of flare‐ups and problems that have been captured in real‐time, thus avoiding the problems of recall.^5^, ^6^, ^10^ The possibility exists for patient care and shared decision‐making to be enhanced through clinicians engaging with contextualised insights about their patients, whose symptoms are captured and displayed in the graphs.^6^ Enhanced understanding of how RA impacts patients' lives over time can illuminate interactions between clinicians and patients,^11^ contributing to improved holistic knowledge.^2^, ^8^ However, there are gaps in comprehending how patients and clinicians interact with technologies and the data generated by them, due to the heterogeneous aspects of implementation, and limited reporting of these elements.^10^, ^12^ In this paper we report an analysis of data collected during a feasibility trial of the REMORA system. It reflects how patients with RA interact with symptom‐tracking data in the context of their disease experiences, and how that data has the potential to inform shared decision‐making in clinical consultations.

## 2 METHODS

Interviews and observations were conducted with patients and clinicians who opted to participate in a feasibility study that took place at two rheumatology outpatient clinics in Greater Manchester. The overall design is an integrated mixed‐methods study combining feasibility performance measures such as rates of recruitment and intervention uptake, with the lived experience of the individuals who supported, participated, or declined participation in the feasibility trial, via interviews and clinical observations.^9^ The feasibility trial is a precursor to a stepped wedge cluster randomised trial that will be conducted at multiple outpatient rheumatology departments across two regions of the United Kingdom.

### 2.1 Recruitment and eligibility

The feasibility study was conducted in two outpatient departments, recruiting adult patients (≥18 years of age) with RA or inflammatory arthritis. Sites were asked to recruit up to 30 eligible patients over a 3 month period. Patients were asked to track their symptoms for up to 6 months using the REMORA app. Patients who had been using the app for at least 18 days and had consented to be contacted were invited to take part in one semi‐structured interview, and offered the choice of in‐person, online, or telephone interviews. Clinicians who had consented to be contacted were invited to take part in a semi‐structured interview, and were offered the choice of in‐person, online, or telephone interviews. All interviews were audio‐recorded on an encrypted digital audio‐recorder and transcribed verbatim by a professional transcription service. Patients who had consented to be contacted were invited to have their consultation during an RA clinic observed by a Nonparticipant observer. Consent was also given by clinicians who agreed to their consultation being observed.

All interviews and observations were conducted by the lead author (SS), a female researcher with extensive experience conducting qualitative in‐depth interviews with a range of child and adult patients and clinicians. The researcher has lived experience of inflammatory arthritis, which informs their understanding of life with a chronic condition and treatment options. The researcher spoke by telephone with each participant before agreeing an interview date and time, she answered any queries they had and explained in more detail the aims/purpose of the study. If appropriate, they explained their interest in the patients' lived experience of RA, such as what life with RA looked like for them. The patient's had a phone and email contact and were invited to ask for any clarifications they had regarding the research and their participation in it.

### 2.2 Data collection

Qualitative semi‐structured interviews were conducted with patients to explore their experiences of using the app, such as functionality issues and of discussing the graphical data with clinicians when attending consultations. Participants were asked open‐ended questions, inviting them to speak in as much or as little detail as they chose about life with RA. Nonparticipant observations of clinical consultations were also conducted to observe how the patient‐clinician dyad used the graphical data during consultations and to explore how shared decision‐making occurs during appointments. All observations were audio‐recorded, and field notes were taken by one observer (SS). Qualitative semi‐structured interviews were conducted with clinicians to explore their experiences of discussing the graphical data with their patients and their thoughts on the management of RA. We conducted interviews with all consented patients and clinicians to ensure that we had a wide range of views regarding their experience, as opposed to ending the data collection period when data saturation occurred.

### 2.3 Data analysis

Transcripts from the interviews and audio‐recording of observations were uploaded to NVivo 12 Plus software, read through at least twice, and checked against audio recordings for fidelity. Thematic analysis^13^ was used to identify and interpret emergent themes from the interviews, with initial coding of topics relating to the impact of RA in patients' lives and how they cope, and patient and clinician thoughts on the symptom tracking data and discussion of it during consultations. Observation recordings were coded using an ethnographic approach, before being triangulated with data from the interviews as themes emerged and were refined. Ethnographic approaches enable the study of various interacting forces upon those living with long‐term conditions such as RA, examining the gap between words and deeds.^14^, ^15^ Themes deriving from the data were discussed by the wider REMORA team during team meetings. In this current paper, the REMORA system is part of an assemblage that also comprises the patient, their lived context and experience of RA, consultations with clinicians, and the broader field of resources patients draw from to cope with their condition.^15^

### 2.4 Ethical approval

Approval for the study was given in April 2022 by the South Central ‐ Berkshire B Research Ethics Committee (ref: 22/SC/0103). All participants gave informed consent to be interviewed and/or observed.

## 3 RESULTS

25 patients who had consented to be contacted to take part in an interview were invited to participate of whom 4 did not respond or declined the invitation. A total of 21 patients living with RA were interviewed, ranging in age from 23 to 80 years old. Most identified as white and British, with one participant identifying as nonwhite. The majority of participants were female (n = 17), aligning with the female preponderance of RA. 7 clinicians were interviewed, 5 female and 2 male. Telephone interviews were opted for by all patients due to their convenience and flexibility, apart from one who chose to be interviewed face‐to‐face. Online interviews using video‐conferencing software were opted for by all clinicians apart from one who chose to be interviewed by telephone. Interviews ranged in duration from 7 to 35 min according to participants’ availability. Some interviews had taken several attempts to arrange due to participants having busy schedules; however, despite their brevity, the interviews provided useful, contextual data. 5 observations by a nonparticipant observer were conducted during consultation appointments between a patient and clinician. Observations ranged in duration from 12 to 36 min. Four patients who were observed also participated in an interview. P refers to Patient interview, C refers to Clinician interview, and OBS refers to observation of patient/clinician dyad consultation.

### 3.1 Living with RA

During interviews, the patients' commentary on tracking their symptoms led to conversations on broader themes, indicating how RA impacts in multiple ways across social, mental, emotional, and physical domains, influencing how patients cope. The initial years of having RA could be challenging to live with:

In the first few years, gosh, it was a real rollercoaster compared to now (P15).

The persistent nature of some patients' pain leads to them being inured to it, based on the realities of limited relief from pain:

I don't know if it's just for me personally but when you're living with a chronic condition, you get used to it and your level of expectation is lower. (P15)

Participants explained the lived experience of having an incurable condition:

… it had a massive impact at the beginning because I couldn't walk, I couldn't get up, I couldn't sit down, just all the time I was in pain. But we got that under control, however it's not really ever gone, and I've not been at a point where I can go, oh yeah, I can just get on with life now. (P04)

But you've just got to pace yourself, I think, and just go with your pain… (P07)

### 3.2 Employment

The demands of staying in employment, despite frequent fatigue and pain, were difficult:

I am now retired. But…I struggled very much when I worked, just getting to and from work was a big issue, because it was mainly in my feet and my ankles at that time, then moved into my hands. (P06)

Having a physically active job is arduous, requiring the support of colleagues, and the patient's adaptation to pain and loss of strength:

… the girls I work with are not too bad. They know [if] I'm having a bad day, they'll carry me… (P03)

… it was murder, when I first had it, it was terrible, at the start, I was going to have to pack in work and all that. Anyway, I'm all right now, I can get through…I notice my strength's gone in my wrists. (P09)

For the youngest participant, in their early 20s, the diagnosis is life‐changing, they managed to complete university, but progressing into employment is not currently viable:

I'm a qualified (healthcare professional) now and I'm not even working, that's how much it's affected my life, because the medication isn't working for me, I'm on my tenth medication now and none of them have worked. (P12)

While some patients are prepared to engage in modifications that enable them to continue working despite pain, sometimes with the help of co‐workers and modification of tasks,^8^, ^11^ for others, employment is no longer manageable:

Well, initially I struggled on at work until I was actually mainly office‐bound … and I found myself just falling asleep at my desk, I couldn't keep awake… then I got the pain in my shoulders which was just completely unbearable, and I had to go on long‐term sick. (P16)

### 3.3 Managing one's RA

The participants had a range of experiences in dealing with RA, frequently demonstrating resourcefulness, fortitude, and determination to help themselves and keep going:

You've got to carry on, you can't not carry on. (P08)

I go to aquacise four times a week, and just try and walk every day, just keep moving, ‘cause it helps. If you don't, you just seize up more. (P05)

I've just, literally, finished counselling… last week. I had to wait 18 months for it. (P12)

A recently diagnosed patient who runs a construction business is working on their strength training:

I have a personal trainer… and we do it, like, with certain exercises where they don't hurt my joints or, you know, I'm not putting strain on my joints. But I'm also building the muscle around the joints. (P20)

Another newly diagnosed patient in their early 30 s is finding the adjustment to RA uncertain; getting a balance between exercise and resting is difficult to judge:

But my big, big worry is how much of a normal life can I live. Even with the Methotrexate, do I still wake up in the morning a bit achy? Do I still get those symptoms?… I think I notice it a lot more because just going to the gym and working out, it's such a big part of what I enjoy. (P21)

A participant who has lived with RA for over 30 years misses out on valued activities:

The biggest issues I would say…I can cope with my pain, I can take tablets. Mine is more social and being able to do things better with my grandchildren. I can't go on long walks… I am lucky if I can struggle to go to the shop. (P17)

Managing one's RA entails taking regular medications; however, issues in the ordering, supply, and side‐effects of drugs can be problematic, causing gaps in medication regimes that have negative effects on patients' wellbeing:

I was laughing on Monday because I phoned the doctors for an emergency prescription which was ready Wednesday, I was laughing with her, I was like, it's not really an emergency prescription but it is what it is! (P09)

… my local pharmacy shut, and we had to change to another one… the pharmacy we moved to was just totally inundated. So, I put a prescription in, and it was 11 days before I actually got it… which sort of sent me in a downward spiral… (P19)

One of the side effects of this new drug is severe hives and itching. I went to see the GP and he said, ‘we don't get involved’. Although it is shared care between your GP and rheumatology, he [the GP] won't step on rheumatology's toes. (P18)

### 3.4 Reviewing the graphical data

Some of the topics and contexts raised by the patients, such as psychosocial factors and lifestyle adaptations can be difficult to initiate in discussions with clinicians. It is feasible, however, that symptom tracking data can open up conversations, with clinicians then learning more about their patients. Participants could see the benefits of their clinician having access to the graphical data:

I think it does help make your face‐to‐face consultations quicker, maybe…and obviously, then they [clinicians] can understand where I'm coming from a lot more. Instead of me feeling like…sometimes I feel like I'm moaning about it all, because there's so much of it… (P07)

When I walked in, she [clinician] said to me, ‘I've got all your data here now, I can see…how you've been going on’. That is brilliant, because it takes the pressure off you, because you don't really have to remember when you've had flare ups… it's all there. (P06)

No, it didn't slow the appointment down. It was just that we could chat about what was going on as well. So, it does help. (P03)

Reviewing the graphical data for the first time was sobering for a patient who was struggling with pain and fatigue both from their RA and another condition:

And I could see the graph… and I thought, oh, gosh, that's me. And then I came away from there thinking, I'm in constant pain, when's it going to ease off… (P07)

Thinking about pain scores was a new experience, and during an observation, a clinician encouraged the patient to focus on their own personal pain scoring:

Patient: You know, everyone has a different experience, don't they, and I see… ten as I'm nearly dead'cause I can't stand the pain. So that's my interpretation. Other people might have a different interpretation, mightn't they?

Clinician: Yeah, but the thing is though, is all this is your data. So, there's nobody else saying, this is what a ten looks like. We know what your one looks like and what your two looks like…(OBSP05)

One patient reported that the clinician did not discuss the symptom tracking data with them during a consultation, despite their expectation this would occur:

I was expecting it to be mentioned but I just said at the end, I said, ‘by the way, I'm doing the REMORA study’, and they said, ‘thanks for your help with that’. But I don't know if they had the information. (P15)

Nevertheless, they conjectured that clinicians may be able to make objective decisions based on pain scoring:

A clinician might be able to look at it more objectively and say, actually, on that combination of medication, we'd expect you up at twos and ones, not at fours and five, actually we do need to do something. (P15)

In another example, a clinician reported that a patient chose not to discuss the data during their consultation:

… I've had one patient who tracked, sort of, like, really religiously but then I really struggled in the consultation to engage her with looking at the data… she wasn't particularly engaged in looking at it with me or really talking about it. So, from a shared decision‐making point of view, obviously we try and let the consultations be patient led. So… I then let her lead the consultation in a different direction. (C02)

In the above case, shared decision making was shaped and acted upon by following the patient's lead and letting their preferences direct the consultation.

During one observation, a clinician became aware of a discrepancy in how a patient was scoring their pain, which did not match with the pain and discomfort they verbally reported, and which was evident in swollen joints during the consultation. When asked about this discrepancy, the patient explained their thinking about pain scores:

… sometimes really it probably could be probably about seven or an eight and I just think, oh, just put one, I'm not that bad. There's people worse off than me… today, my hands are sore, but tonight, I will still only score at one on my [app]… (OBSP03)

The clinician took the opportunity to use this discrepancy by coaching the patient to be realistic about their pain and to use anti‐inflammatories regularly, rather than sporadically. They also explained the possibility of trying a different range of drugs to manage the pain and inflammation:

… when I look at your hands, you're quite stoical in respect of, ‘it's all right, I'm managing’…. But… if we've got inflammation in a few areas, we can get you on a different group of medications. (OBSP03)

As in the above interaction, patients with RA can be stoic, normalising discomfort, desiring to be seen as compliant and minimising their complaints:

… there is definitely that stoical group of patients that will come to clinic once a year or every 6 months and will say that they are fine. (C01)

Patients' under‐reporting of symptoms can be addressed through referring to the graphical data:

Sometimes those conversations are hard to start, whereas the data may help lead that conversation… (C01).

Constructive interactions stemming from reviewing the data act as a two‐way exchange of information, while also informing or ‘challenging’ decisions:

So I've seen from the patients who have engaged with it, it's either very much supported the decision making or sometimes challenged the decision making. And that's equally valuable, I think, to explore the nuances. (C02)

Some patients reported being unsure about scoring pain if it might be caused by conditions such as osteoarthritis or injury; in an interview, a clinician suggested that this was not so important to them as treating the whole patient:

… beliefs and attitudes… maybe influence how they present themselves to us because perhaps we're thinking holistically, but the patient's actually compartmentalising things and thinking in advance, what is it that the clinician wants to know about me at this appointment? (C02)

Meaningful consultations where clinicians are approachable and prepared to listen can encourage patients to reciprocate, resulting in more effective communication that supports continuity of care, which is essential for good management of RA.

## 4 DISCUSSION

It is proposed that clinicians should be curious about those they care for,^16^ and this paper indicates how the graphical data can be used to generate meaningful knowledge about, and with patients. Participants in the study described how they coped when they first got ill, their experiences of living with RA, managing pain and employment, and related experiences. Observations of consultations revealed patients' interactions with clinicians, and the contexts in which shared decision making emerges. The role of clinicians is important in the lives of RA patients,^17^ they act as gateways to critical resources including treatment, pain management and support. During observations it was apparent that patients frequently reported and sought advice on pain management and associated issues, with their clinicians providing careful explanations about test results, the side‐effects of medication, and the possibility of trying different drugs. Fair^1^ commented that when patients were given clinical education and help, the burden of their disease decreased, with patients benefitting from well‐targeted advice and information provided in appropriate, non‐judgemental ways. Some patients may be comfortable with their clinician taking the lead in decision‐making and resist the ‘expert’ patient label,^17^ nevertheless, the clinician's role as an active listener remains key to effective consultations with all their patients.^18^, ^19^

### 4.1 Implications for shared decision‐making

Shared decision‐making emerged at a similar time to patient‐centred care and the biopsychosocial model,^20^ and the guiding principles of these models are based on accepting that an individual's self‐determination is a desirable goal.^19^ This is enacted through building good relationships that uphold autonomy, respecting individual competence and the interdependencies and relational autonomy most people live within.^19^, ^21^ Hence, shared decision‐making involves contextualised decisions,^20^ formed through judgements of what an acceptable burden might be for patients,^22^ and guided by an understanding of their preferences. The data from this study suggests use of the symptom tracking data enables clinicians and patients to explore and inform shared decisions. These are shaped by the patient's wider contexts encompassing employment and financial status, sociocultural context, caring responsibilities, and access to supportive measures such as physiotherapy and mental health support.^23^ Hence, this deliberative process is not strictly rational and may deal with uncertainties emerging from a distributed field of possibilities and influences.^7^, ^20^, ^24^

Shared decision‐making is a point at which patients' agency can be supported, addressing options for medication and treatments that are guided by individual priorities and experiential knowledge.^11^, ^17^ This approach opens up ways of supporting patients emotionally^8^ as well as physically in the task of living with RA. The habituation to discomfort^1^, ^18^ and tendency to under‐report pain, or feel that they are complaining is observed among RA patients.^5^ This resistance to mentioning pain became apparent during an observation, where the patient was encouraged to be more realistic about pain scoring of their swollen joints. This new insight led to the clinician working with the patient to explore the possibility of a revised treatment plan suited to their needs. An earlier phase in the REMORA programme tested out a proof‐of‐concept of the REMORA system,^5^ and it was observed that the graphical data changed the nature of the consultation.^6^ Similarly in this study, the patient and clinician negotiated the meaning of the data, reaching a shared decision to pursue new options.

The patients' symptom tracking data is factored into decision‐making on treatment options as part of a process of collaboration, equipping patients to cope in their specific lived context.^20^, ^25^ This process shifts care away from paternalistic practices,^22^ to one of clinicians working alongside patients to reach a consensus on symptom management suited to them. Self‐efficacy and optimism have beneficial effects on RA patients,^1^, ^26^ and engendering these through paying attention to patients' data and their associated needs align to the values associated with shared decision making.

### 4.2 Limitations

The study was a limited roll out of the REMORA system across 2 new sites, so there were limitations in terms of size and diversity of the patient sample and number of clinicians available for interview and observation. The low number of observations was due to a limited number of both patients and their designated clinicians consenting to being observed, hence even if a patient consented to an observation, their clinician may have declined consenting to observation, and vice versa. Among the clinicians who responded to an interview invitation there were limited experiences of using the graphical data during consultations, with clinicians reporting that some of their patients had failed to track data, had only used the app sporadically, or had led the discussion to other concerns. Likewise, patients reported that clinicians did not always look at or refer to the data during consultations, despite their expectations that this would occur. This outcome limited some patients' ability during interviews to explain their experiences of discussing the graphical data with their clinician. There are some limitations to telephone interviews, in that this restricts the ability to see or react to body language during the data collection. Some of the interviews were very short, limiting the amount of data available for analysis related to the use of REMORA.

## 5 CONCLUSION

As part of this feasibility trial, patient and clinician responses to using and interpreting tracked symptom data from the REMORA system has been explored. Patients and clinicians acknowledged that the graphical data had the potential to bring new knowledge and insights on RA to the fore, as patients are prompted by the data to recall how their condition has been since the last consultation. One of the putative benefits is in opening up conversations where the clinician has the opportunity to act as an educator, partner, and supporter. While the graphical data assists in the recall of flare‐ups and produces graphs of pain and stiffness, it can also be a tool that assists the patient‐clinician dyad in the process of two‐way learning about the task of managing a chronic condition. Symptom tracking apps, such as the one used in REMORA, often elicit a defined field of possible responses, however, with skilful questioning, clinicians can use the data as a basis for well‐targeted discussions that generate meaningful decision‐making. The forthcoming REMORA stepped wedge cluster randomised trial will test out and refine these early findings on a larger and more diverse scale, likely providing more detailed insights on how patients and clinicians engage in shared decision making and how graphical patient‐reported symptom data enhances this process.

## CONFLICT OF INTEREST STATEMENT

WGD has received consultancy fees from Google, unrelated to this work. All other authors have no conflicts of interest to declare.

## Supporting information

Supporting information.

## Data Availability

The data that support the findings of this study are available on request from the corresponding author. The data are not publicly available due to privacy or ethical restrictions.
